# Synergistic Interaction of Phytohormones in Determining Leaf Angle in Crops

**DOI:** 10.3390/ijms21145052

**Published:** 2020-07-17

**Authors:** Xi Li, Pingfan Wu, Ying Lu, Shaoying Guo, Zhuojun Zhong, Rongxin Shen, Qingjun Xie

**Affiliations:** 1 State Key Laboratory for Conservation and Utilization of Subtropical Agro-Bioresources, Guangdong Provincial Key Laboratory of Plant Molecular Breeding, South China Agricultural University, Guangzhou 510642, China; 20183137023@stu.scau.edu.cn (X.L.); 201713070123@stu.scau.edu.cn (P.W.); luying@stu.scau.edu.cn (Y.L.); syguo@scau.edu.cn (S.G.); 2 Guangdong Laboratory of Lingnan Modern Agriculture, Guangzhou 510642, China; zhongzhuojun@stu.scau.edu.cn; 3 State Key Laboratory for Conservation and Utilization of Subtropical Agro-Bioresources, College of Life Sciences, South China Agricultural University, Guangzhou 510642, China

**Keywords:** Leaf angle, Phytohormones, crop yield, BR, Crosstalk

## Abstract

Leaf angle (LA), defined as the angle between the plant stem and leaf adaxial side of the blade, generally shapes the plant architecture into a loosen or dense structure, and thus influences the light interception and competition between neighboring plants in natural settings, ultimately contributing to the crop yield and productivity. It has been elucidated that brassinosteroid (BR) plays a dominant role in determining LA, and other phytohormones also positively or negatively participate in regulating LA. Accumulating evidences have revealed that these phytohormones interact with each other in modulating various biological processes. However, the comprehensive discussion of how the phytohormones and their interaction involved in shaping LA is relatively lack. Here, we intend to summarize the advances in the LA regulation mediated by the phytohormones and their crosstalk in different plant species, mainly in rice and maize, hopefully providing further insights into the genetic manipulation of LA trait in crop breeding and improvement in regarding to overcoming the challenge from the continuous demands for food under limited arable land area.

## 1. Introduction

To overcome the challenge of ever-increasing global demands for food, feedstock, and bioenergy products, breeders have been forced to select and breed cultivars with a key feature that can be planted at higher densities in order to increase grain yield with the limited availability of arable land area [1,2]. To this end, genetic improvement of crops with ideal plant architecture is considered as one of the most powerful strategies for addressing this issue. The key components of ideal plant architecture in crop generally include plant height, grain architecture, and leaf angle (LA) [3]. Since the 1960s, genetic engineering of decreasing plant height in crops has dramatically boosted the crop yield and definitely benefited millions of people worldwide, which is remarked as the “Green Revolution”. The great achievement of Green Revolution is attributed to the utilization of two “Green Revolution” genes, mutant allelic *Semi-dwarf1* (*Sd1*) in rice, which encodes a key enzyme in the gibberellic pathway GA20ox2, and *Reduced height-1* (*Rht-1*) in wheat that encodes a key repressor in gibberellin signaling pathway called DELLA, are responsible for gibberellin metabolism in rice and wheat [4,5], respectively. However, excessive application of nitrogen fertilizer for ensuring the yield and productivity of the semidwarf varieties has brought severe contamination on environment. Recently, a novel gibberellin-GIBBERELLIN INSENSITIVE DWARF1 (GID1)-NITROGEN-MEDIATED TILLER GROWTH RESPONSE 5 (NGR5) signaling pathway has been stated [6], which can be effectively used to boost yield of semi-dwarf crop by simultaneously enhancing the tiller number and nitrogen use efficiency (NUE) in next-generation Green Revolution. Besides plant height, the LA trait has also been substantially selected for high-yield varieties breeding, particularly in maize and rice, due to its vital role for optimal light interception and competition between neighboring plants in natural settings.

**Figure 1:**
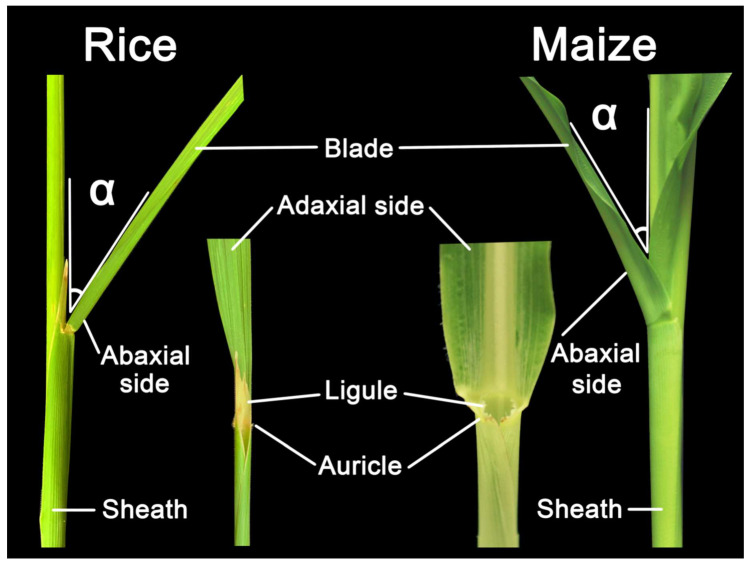
Structural composition of leaf angle in rice and maize. Leaf angle is defined by the angle between the plant stem and leaf adaxial side of the blade, indicating as the α in the figure. This leaf morphology is affected by the development of ligule and auricle as well.

A typical grass leaf is consisted of distal blade, proximal sheath, and a boundary called ligular region (also called lamina joint) that separates blade and sheath into distinct parts, all of which contain epidermal, ground, and vascular tissues that are continuous with each other but distinct in cell types and patterns [7,8]. LA, defined as the angle between the stem and adaxial side of the blade (Figure 1), is one of the most important architecture traits selected for crop yield improvement [9]. Crops with architecture of smaller LA and more upright leaves can facilitate higher plant density and enhance the photosynthetic efficiency, thus elevating yield [10,11,12]. The ligular region, an annular structure outside the joint of the leaf blade and leaf sheath, is the pivotal structure for determining LA in grasses. Accumulating evidences have been implicated that the formation of LA is mediated by the shape of the lamina joint, differences in cell numbers/size at adaxial/abaxial region and distinctive mechanical tissue strength [9,13,14,15], and thus any influence on them could alter the LA. For example, failure of longitudinal elongation of the adaxial cells in the lamina joint may result in erect leaves [16], whereas excessive expansion of the adaxial cells would increase the LA [17,18].

Given the rapid developments of plant functional genomics, a number of genes controlling LA have been cloned and the relevant regulatory network underlying the development of LA in grasses has been well characterized. These studies revealed that phytohormones, such as brassinosteroids (BRs), auxin, and gibberellins (GAs), comprehensively participate in regulating LA by orchestrating the homeostais of their biosynthesis and the expression of signaling transcription factors (TFs). Though each phytohormone and TF has been found to contribute quite similar traits/phenotypes in term of LA, how they interplay with each other is still far beyond understood. This review is an attempt to highlight the regulation mechanism of LA and the genetic interactions among phytohormones in regulating LA formation with particular emphasis on maize and rice.

## 2. Regulation of Lamina Joint Bending by Brassinosteroid (BR)

**Figure 2:**
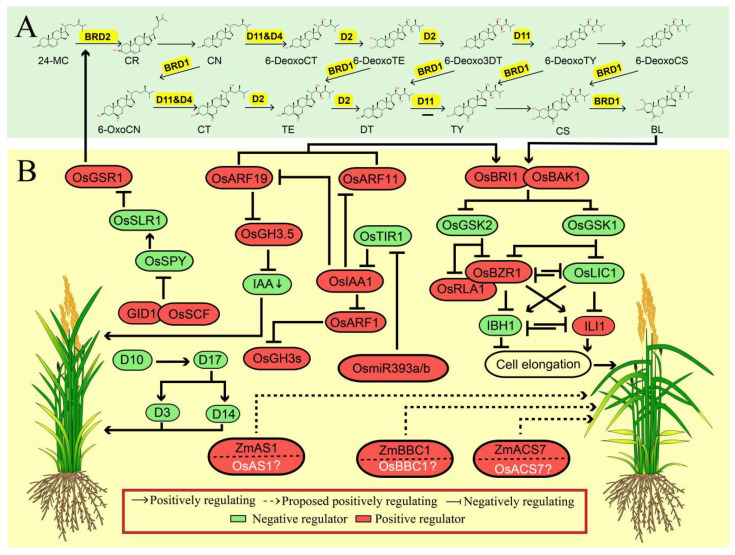
The synergistic regulation mechanisms of leaf angle by phytohormones in rice. (**A**) Brassinosteroid biosynthesis pathway. The corresponding enzyme that catalyze each reaction in yellow color. The secondary structure of the chemical is obtained from ChemSpider (http://www.chemspider.com/Default.aspx). (**B**) Positive and negative regulation of leaf angle by brassinosteroid (BR) signaling pathway and its crosstalk with other regulators involved in other plant hormones pathway, and other phytohormones regulators positively participate in regulation of leaf angle. The proteins in white color and with a question mark represent that they are homologs of those reported to be involved in the regulation of leaf angle. The green arrow represents the downregulation of indoleacetic acid (IAA). The protein in red color box represents as the positive regulator while the one in green color box represents the negative regulator of leaf angle. The dashed line and solid line represent the indirect or direct evidence supporting the responsible regulation, respectively.

BRs are a group of steroid phytohormones involved in many important biological processes, and thus play a vital role in regulating several important agronomic traits, such as leaf angle, plant height and stress resistance. The regulatory pathways of BR biosynthesis, metabolism, and signal transduction have been well established in rice [19,20,21]. Since LA is a grass-species-specific trait, the role of BR in regulating LA has only been characterized in maize and rice rather than Arabidopsis, indicating that BR is a positive regulator of LA (Figure 2).

Up-regulation of the BR content within lamina joint region or enhanced BR signaling pathway by boosting the expression of BR related regulators resulted in increasing LA (Table 1). For instance, many researches in rice have elaborated that leaf inclination is closely associated with biosynthesis or signaling of BR [19,22]. Loss-of-function of BR biosynthetic genes, such as *Dwarf 2* (*D2)*, perturbed the endogenous level of BR, eventually resulting in erect leaf phenotype [11,23,24,25,26]. Besides, other BR biosynthetic regulators, such as the Cytochrome P450 family proteins, have also been documented to function in the development of LA. For example, *BR-deficient Dwarf1* (*OsBRD1*) is cytochrome P450 protein and encodes a key enzyme (BR C-6 oxidase) catalyzing BR biosynthesis. Disruption of *OsBRD1* causes pleiotropic effects, including severe dwarf phenotype, tortuous leaves, short panicles, small seeds, etc. [27,28]. Another two Cytochrome P450 proteins, OsDWARF4 and OsDWARF11, also catalyze the rate-limiting reaction (c-22 hydroxylation) of BR biosynthesis. *OsDWARF4* is highly expressed in leaf blade and root, which is inhibited by BR but increased in BR-insensitive or -deficient mutants, indicating there is a feedback regulation on *OsDWARF4* expression. Depletion of *OsDWARF4* caused mild phenotype with erect leaves but not any detrimental effect on the development of leaf, inflorescence, and seed relative to wild-type plant [11,24]. Distinct from *Osdwarf4* mutant, *Osdwarf11* mutant showed a much more severe phenotype, including dwarfism in plants, erection of leaves, pollen abortion, and small grains. Further investigation revealed that *OsDWARF4* and *OsDWARF11* function redundantly in BRs biosynthesis, but *OsDWARF11* performs a major role in BR biosynthesis pathway while *OsDWARF4* plays the complementary role [25], which explained the different effects of them in term of plant growth and development, as well as the LA. It is worthy to mention that the *Osdwarf4* mutant with erect leaf simultaneously promoted biomass production and higher yields than wild type at different planting densities, even without additional fertilization, indicating that this gene/allele is a potential candidate for sustainability increasing crop yield in limited land area [11]. Taken together, these studies have clearly illustrated that BR metabolism is responsible for the LA formation in crop.

In the BR signaling transduction pathway, BR is perceived by extracellular domain of BRASSINOSTEROID INSENSITIVE 1 (BRI1), a single transmembrane leucine-rich repeat receptor-like protein kinase (LRR-RLK), and its co-receptor BRI1-ASSOCIATED KINASE 1 (BAK1). In the absence of BR, BRI1′s kinase domain is deactivated by the negative factor BRI1 KINASE INHIBITOR 1 (BKI1); while BR is present, the BR compound binds to the extracellular domain of the BRI1 and BAK1. Subsequently, BRI1 phosphorylates BKI1, which results in the release of BKI1 from the plasma membrane, and then induce the phosphorylation of the kinase domain of BRI1 and BAK1, finally activating the initiation of BRI1-mediated signaling transduction [29]. The activation of BRI1 in turn phosphorylates downstream BR-SIGNALING KINASE1 (BSK1), CONSTITUTIVE DIFFERENTIAL GROWTH 1 (CDG1), and some of their homologs. Both BSK1 and CDG1/CDL1 phosphorylates BRI1-SUPPRESSOR 1 (BSU1) and subsequently activates BSU1. Activated BSU1 dephosphorylates and inactivates BRASSINOSTEROID INSENSITIVE 2 (BIN2) to release the suppression of BRASSINAZOLE-RESISTANT 1 (BZR1) and BRI1-EMS-SUPPRESSOR 1 (BES1), which are two key downstream transcription factors positively mediating BR responses. BZR1 and BES1 are rapidly dephosphorylated by Protein Phosphatase 2A (PP2A) family, leading to their nuclear accumulation and their regulation of thousands of BR-responsive genes expression [30]. Besides, in the absence of BR, the interaction of phosphorylated BZR1 and BES1 with 14-3-3 proteins, a group of conserved phosphopeptide-binding proteins, can lead to their cytoplasm retention and degradation, while in the presence of BR, dissociated BKI1 in cytoplasm can competitively bind to 14-3-3 proteins to inhibit this process [31]. Otherwise, the PPA2 dephosphorylates BRI1, BZR1 and BES1, relieving the binding of BZR1 and BES1 to 14-3-3 proteins, which regulates downstream genes in response to BR and induce diverse BR responses [32]. In addition to the BR biosynthetic genes mentioned above, BR signaling genes also play an essential role in regulating the development of LA in rice. For example, OsBRI1 is an ortholog of Arabidopsis BRI protein in rice. *OsBRI1* participates in regulating various aspects of growth and development processes in rice, including the bending of lamina joint, the intercalary meristem formation, and the longitudinal elongation of internode cells. Therefore, depletion of *OsBRI1* significantly perturbed the internode elongation and bending of the lamina joint [33]. *OsBAK1* (a homologous gene to *Arabidopsis BAK1*) encodes SERK family receptor kinase protein and acts as co-receptor kinase for OsBRI1 to mediate BR signal transduction. Down-regulating the expression level of *OsBAK1* produced a rice variety with erect leaf and normal reproduction so that considered as a promising target for improving rice grain yield [34]. Nevertheless, suppression of *OsBZR1* expression by RNA interference (RNAi) in rice greatly altered the expression of BR-responsive genes and resulted in plant dwarfism and erect leaf, suggesting that *OsBZR1* plays an central downstream role in BR signaling [35]. The rice 14-3-3 proteins have been proven to interact with and retain OsBZR1 in cytoplasm instead of nucleus, ultimately leading to inhibiting the function of *OsBZR1*. However, BR treatment can dissociate their interactions and activate *OsBZR1*, in turn causing the erect leaf phenotype [36]. Similarly, a rice zinc finger transcription factor, *LEAF* and *TILLER ANGLE INCREASED CONTROLLER* (*OsLIC*)*,* also antagonized *OsBZR1* to repress BR signaling in rice. Overexpression of *OsLIC* resulted in erect leaves by eliminating BR response, indicating that *OsLIC1* negatively modulates leaf inclination in rice. *OsLIC* directly regulated the *INCREASED LEAF INCLINATION 1* (*ILI1*), a positive regulator in lamina inclination, to oppose the action of BZR1 [36]. In addition, recent studies have identified an APETALA2 (AP2)/ERF (ethylene-responsive element binding factor) family transcription factor, *Reduced Leaf Angle 1* (*RLA1*) that is identical to the *SMALL ORGAN SIZE 1* (*SMOS1*), as a positive regulator of BR signaling, which physically interacted with OsBZR1 to enhance its transcriptional activity to enlarge LA in rice [37,38]. *BU1* encodes a helix-loop-helix protein and acts as a novel BR positive regulator. Overexpressing *BU1* in rice led to enhanced bending of the lamina joint, increased grain size, and resistance to BR, while repression of *BU1* and its homologs in rice displays erect leaves [39]. Furthermore, a pair of antagonizing HLH/bHLH factors, *OsILI1* that is the homology of *Arabidopsis thaliana Paclobutrazol Resistance 1* (*PRE1*) and *ILI1 binding bHLH 1* (*OsIBH1*), functioned as downstream factors of *OsBZR1* to regulate leaf angle. *OsILI1* positively regulated BR-mediated cell elongation, whereas OsIBH1 directly interacted with OsILI1 and performed an opposite role [40]. In addition, another bHLH transcription factor, *BRASSINOSTEROID-RESPONSIVE LEAF ANGLE REGULATOR 1* (*OsBLR1*), has also been implicated to participate in LA regulation though the BR pathway in rice. Over-expressing *OsBLR1* simultaneously increased leaf angle, grain length and sensitivity to BR, whereas mutation of *OsBLR1* resulted in erect leaf and shorter grain [41]. Similarly, gain-of-function of *OsbHLH079* also caused wide LA, longer grain and hypersensitive to BR in rice, while *OsbHLH079*-RNAi lines showed opposite phenotype [42]. Collectively, these findings suggested that *bHLH* family transcription factors may be broadly involved in BR-mediated LA regulation. Previously, a plant-specific gene family transcription factor, *OsGRAS19* (*GA INSENSITIVE (GAI), REPRESSOR OF GAI (RGA),* and *SCARECROW (SCR) 19*) was suggested to be involved in regulating BR signaling, because the knockdown lines of *OsGRAS19* displayed less sensitivity to the 24-epi-brassinolid (BL) treatment as compared to WT in rice. Higher expression of *OsGRAS19* caused larger LA, narrow leaf and thin culm and panicle in rice [43]. Recently, a novel mutant of *OsGRAS19*, *D26*, has been identified in rice, which displayed typical BR-mediated phenotypes, including semidwarf, wider, and shorter leaf in addition to the erect leaf, as well as longer grain [44]. Taken together, these results further elaborated that the BR signaling is extensively involved in regulating LA in rice.

Until now, the molecular regulation of BR underlying LA in maize has been rarely investigated. Previous reports illustrated that suppression of *ZmBRI1* or knock-out of *Dwarf* and *Irregular Leaf 1* (*ZmDIL1*) displayed dwarf plant and erect leaves [45,46], suggesting a conserved function of BR for plant architecture in monocots. Recently, a SQUAMOSA-PROMOTER BINDING PROTEIN-LIKE (SPL) family protein LIGULELESS 1 (LG1), which is recognized as a conserved key factor in the formation of ligule and auricle in both rice and maize [47,48], confers an important role in controlling the leaf angle through regulating BR and auxin signaling pathway in maize and wheat. In maize, *ZmLG1* activated a B3-domain containing transcription factor *ZmRAVL1*, which sequentially activated *ZmBRD1* that was also designated as *Upright Plant Architecture 1* (*UPA1*), eventually resulting in the alternation of leaf angle. *UPA2* functioned as a distant *cis* element to regulate *ZmRAVL1* expression, which was controlled by the *Drooping Leaf 1* (*DRL1*). This *UPA2*-*ZmRAV1*-*UPA1* module fine-tuned the BR pathway and finally determined the plant architecture and LA in maize [49]. In wheat, the *LG1* ortholog, *TaSPL8*, directly bound to the promoter of an auxin response factor *TaARF6* and the BR biosynthesis gene *CYP90D2* (*TaD2*), and subsequently activated their expressions, leading to enhancing lamina joint development [50]. Recently, *ZmILI1,* an ortholog of *OsILI1* that plays an important role in LA in rice, was found to directly bind to the *ZmLG1* and *CYP90D1* promoters to affect the BR biosynthesis and signal, eventually changing the LA [51]. Collectively, these studies supported a notion that components of BR signaling pathway play a dominant and conserved role in the formation of leaf angle in multiple crops and would be the potential targets for genetic improvement of crop architecture and yield.

## 3. Regulation of Lamina Joint Bending by Indoleacetic Acid (IAA)

Indoleacetic acid (IAA) is another crucial hormone, regulating leaf inclination mainly through patterning the adaxial/abaxial cell growth of leaves. In contrast to the positive role of BR in LA regulation, IAA functioned as a negative regulator since eliminating IAA content resulted in increased leaf inclination while increasing IAA content caused reduction of leaf inclination and upright leaves [52].

Auxin signal transduction is a sophisticated pathway, which is consisted of several key components, including the F-box TRANSPORT INHIBITOR RESPONSE 1/AUXIN SIGNALING F-BOX PROTEIN (TIR1/AFB) auxin co-receptors, the Auxin/INDOLE-3-ACETIC ACID (Aux/IAA) transcriptional repressors, and the AUXIN RESPONSE FACTOR (ARF) transcription factors and a ubiquitin-dependent protein degradation system. When IAA is deficient, Aux/IAA proteins form heterodimers with ARFs and block the function of ARFs to inhibit the auxin signal. Presence of auxin promotes the interaction between TIR1/AFB and Aux/IAA proteins, and then triggers a proteasome-mediated degradation of Aux/IAA by 26S Proteasome, eventually resulting in the release of ARF transcriptional activity. Subsequently, activation of ARF induces the changes of auxin-mediated gene expression pattern and growth responses [53,54,55,56]. This auxin signaling transduction model in rice has been implicated to associate with the regulation of lamina joint bending (Figure 1).

For instance, *FISH BONE* (*FIB*) encodes an orthologous of TAA protein, which plays a negative effect on leaf inclination. Loss-function of *FIB* caused a reduction of IAA level and altered auxin polar transport activity, thus producing small leaves with enlarged lamina joint angle [57]. *LEAF INCLINATION1* (*LC1*) encoding an IAA amino synthetase, termed as OsGH3.1 in rice, maintained auxin homeostasis by catalyzing excess IAA binding to various amino acids. A gain-of-function mutant *lc1-D* in rice displayed a reducing content of free IAA and increasing leaf angle due to the promotion of cell elongation in the adaxial surface of lamina joint [58]. In addition, two *OsMIR393a/b* targeted auxin receptors, *OsTIR1* and *AUXIN SIGNALING f-box 2* (*OsAFB2*), have also been proven to be involved in the regulation of leaf angle. Overexpression of *OsmiR393a/b* repressed the expression of *OsTIR1* and *OsAFB2*, which led to greater leaf angle in rice [59]. Further study demonstrated that BR promoted *OsTIR1* and *OsAFB2* to trigger the degradation of OsIAA1, which resulted in de-suppression of OsARF11 and OsARF19 proteins and consequently caused enlarge LA in rice [60]. Furthermore, a SPOC domain-containing transcription suppressor Leaf inclination 3 (LC3) regulated leaf inclination through interacting with a HIT zinc finger domain-containing protein, LC3-interacting protein 1 (LIP1). Meanwhile, LC3 could also directly bind to the promoter regions of *OsIAA12* and *OsGH3.2* to regulate auxin signal transduction and auxin homeostasis, finally influencing the formation of leaf inclination [61]. 

The maize auxin efflux carrier P-glycoprotein (ZmPGP1) was an adenosine triphosphate (ATP) binding cassette (ABC) transporter, which was involved in the polar transport of auxin and associated with the LA in maize [62]. Notably, multiple haplotypes of *ZmPGP1* were present in various landraces, teosintes, and inbred accessions [63], suggesting a domesticated selection and improvement due to the demand of higher density planting. 

## 4. Regulation of Lamina Joint Bending by Gibberellins (GA)

GA signaling pathway has also been implicated to participate in the regulation of the LA through the BR signaling dependent manner. Previously, transcriptomic profiling identified the co-expression pattern between GA and BR associated genes, however, it was less understood whether these genes were coordinated to regulate the LA. Recently, several researches have verified that certain GA genes interact with BR genes to modulate the LA. For instance, knockdown of the rice *SPINDLY* (*OsSPY*) caused the eliminated expression of BR biosynthesis genes *D11*, *D2*, and *OsDWARF*/*BRD1*, but increased the *OsDWARF4* expression that functions downstream of the GA gene, *SLENDER RICE 1* (*SLR1*), ultimately resulting in the enhanced leaf angle [64]. A recent study found that the Arabidopsis O-fucosyltransferase SPY could mono-O-fucosylate the DELLA protein, leading to higher affinity of interaction between DELLA and BZR1 [65]. From this point of view, it is supposed that OsSPY also could activate the OsSLR1 encoding a DELLA protein to interact with OsBZR1, thereby affecting LA in rice. The rice GA-stimulated transcript gene (*OsGSR1*) induced by GA is another downstream gene of *SLR1*, and it directly binds to BRD2 to enhance the BR biosynthesis, which subsequently altered the leaf angle [64,66]. In addition to these GA genes, other regulators involved in GA pathway have also been identified to participate in LA regulation. *OsDCL3a* encodes a Dicer-like endoribonuclease involved in generating siRNA. Down-regulation of the *OsDCL3a* resulted in increased leaf angle by modulating the expression of GA and BR associated genes, including *OsGSR1* and *BRD1* [67]. Recently, *OsmiR396d* has been reported to regulate the LA in rice, which was promoted by OsBZR1 and then regulated the expression of BR responsive genes by targeting the *GROWTH REGULATING FACTOR 4* (*OsGRF4*) [68]. Alternatively, as another target of *OsmiR396d*, the *OsGRF6* participated in GA biosynthesis and signal transduction but was not directly involved in BR signaling only modulated the plant height rather than LA [68]. Taken together, it is supposed that GA likely regulates LA dependent on the BR pathway, and thus identification of much more GA components involved in response to BR would extend our knowledge to this issue. 

## 5. Regulation of Lamina Joint Bending by Crosstalk among Various Phytohormones

Recent studies have shown that crosstalk between IAA and BR cooperatively regulates the development of leaf angle. OsIAA1 is a key negative regulator for auxin signal transduction, which is induced by auxin and BR. Over-expression of *OsIAA1 i*n rice resulted in dwarfism and increased leaf angle with decreased sensitivity to auxin treatment but increased sensitivity to BR treatment. *OsARF1* is an auxin signal positive regulator which is inhibited by *OsIAA1* in the absence of auxin. Further analysis showed that mutation of *OsARF1* reduced sensitivity to BR treatment, resembling the phenotype of *OsIAA1*-overexpression plants, which indicated that BR may interact with auxin through the OsIAA1-OsARF1 module to regulate LA in rice [69,70]. *OsARF19* acted as another coordinator of auxin and BR by positively regulating the expression of *OsGH3.5* to reduce the content of free IAA on the one hand and activating *OsBRI1* to stimulate BR signal cascades on the other hand, thus resulting in increased leaf angle [71]. *RLA1*/*SMOS1* functioned downstream of the auxin signaling pathway, and enhanced the transcriptional activity of *OsBZR1* by interacting with OsBZR1, suggesting that *RLA1*/*SMOS1* integrated BR and IAA signal pathway to regulate the development of leaf angle [37,38]. In summary, the above studies showed that the auxin antagonized BR by interfering both BR metabolism and signaling to negatively regulate the leaf angle in rice.

Additionally, the rice *d1* mutant with null function of an α subunit of G-protein (Gα), Rho GTPase activating protein 1 (RGA1), exhibited a dwarfism phenotype with erect leaves, and reduced sensitivity to GA and BR, indicating that *RGA1* was involved in both GA and BR responses [29,72]. Further studies showed that D1/RGA1 interacted with TUD1 to induce *BU1* expression, resulting in the increased leaf inclination [73,74]. BR can also act upstream of GA by modulating GA metabolism to regulate cell elongation. BR activated *OsBZR1* and induced the expression of *D18*/*GA3ox-2*, one of the GA biosynthetic genes, leading to increased bioactive GA levels in rice seedlings. In contrast, GA extensively inhibited BR biosynthesis and the BR response with a feedback mechanism, so that GA treatment decreased the enlarged leaf angles in plants by attenuated BR biosynthesis or signaling [75]. These results showed that BR and GA were intertwined to regulate the leaf angle in rice.

## 6. Other Phytohormones Involved in Regulation of Lamina Joint

**Figure 3:**
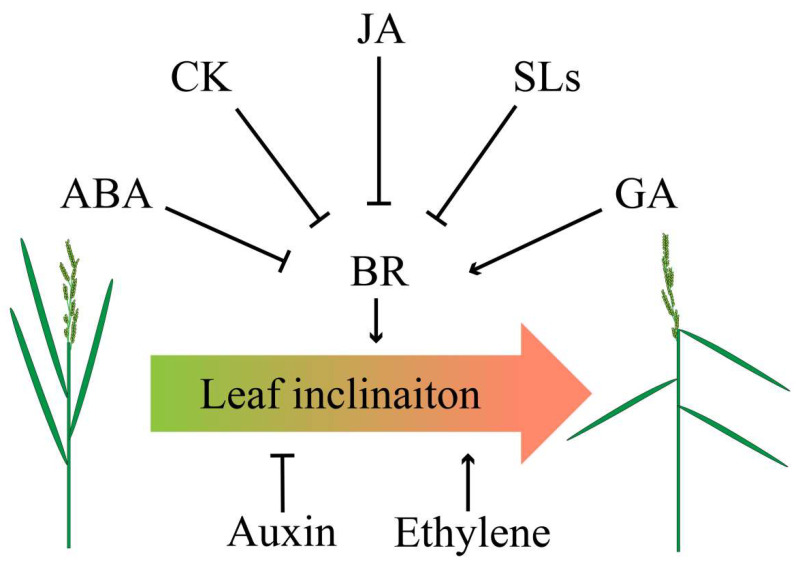
Crosstalk of phytohormones in determining leaf angle. Abscisic acid (ABA), CK, jasmonic acid (JA), and strigolactones (SLs) negatively regulate BR, thereby inhibiting leaf angle, whereas gibberellins (GA) positively coordinates BR to increase leaf angle. Application of auxin results in erect leaf while ethylene leads to flat leaf.

Phytohormones such as ethylene, strigolactones (SLs), jasmonic acid (JA), and abscisic acid (ABA) were also involved in regulating leaf inclination of rice (Figure 3). An early report demonstrated the interaction of ethylene and BR to regulate leaf inclination of rice, but the underlying mechanism remaining to be elusive [18]. Currently, a study has validated that altering the C terminus of 1-Aminocyclopropane-1-carboxylate (ACC) synthase 7 (ZmACS7) responsible for ethylene biosynthesis in maize led to the stability of this protein and the accumulation of ACC and ethylene contents, as well as the up-regulation of ethylene responsive genes, which finally reduced plant height and increased leaf angle [76]. Similar to *ZmACS7*, overexpression of its closest paralog *ZmACS2* also resulted in flatter leaves [76], further suggesting that ethylene positively regulates LA.

SLs negatively regulate leaf inclination at seedling stage [77]. Interestingly, recent report showed that SLs also mediated leaf inclination in response to nutrient deficiencies in rice [78]. Similar to SLs, JA also showed a negative role in leaf inclination. JA treatment decreased lamina joint inclination by repressing the expression of BR biosynthesis-related genes which thus decreased endogenous BRs levels. Besides, inactivation of a negative regulator of BR signaling, GSK3-like kinase, partly rescued the inhibited effect of JA on lamina joint inclination, indicating that JA may disturb both BR biosynthesis and BR signaling pathway to limit lamina joint inclination [79]. 

Previous research has revealed that BR antagonized with ABA in regulating seed germination and hypocotyl elongation in Arabidopsis. The study showed that the ABSCISIC ACID INSENSITIVE5 (ABI5) directly interacts with BRASSINOSTEROID INSENSITIVE2 (BIN2), and then was phosphorylated and stabilized by BIN2 upon ABA treatment [80]. Additionally, the BES1 also physically interacted with ABI5 to hinder the expression of ABI5-targeted *EARLY METHIONINE-LABELED 1* (*EM1*) and *EM6* [81], eventually facilitating the seed germination in Arabidopsis. Interestingly, the ABI1 and ABI2 can interact with and dephosphorylate BIN2 to attenuate the BR signaling in Arabidopsis [82], indicating a complicated crosstalk between ABA and BR. Recently, there is research which further revealed that ABA also antagonized BR to regulate the lamina joint inclination in rice by targeting the BR biosynthesis gene *D11* and BR signaling genes *GSK2* and *DLT* [83], and it therefore raises an issue that whether the rice homologs of Arabidopsis *ABI1* and *ABI2* may also interfere the BR-mediated LA. Investigation of the LA phenotype of the *Osabi1* and *Osabi2*, as well as overexpression lines of these two genes, may provide the answer for this hypothesis. Another research preprinted in bioRxiv demonstrated that a transcriptional repressor *ZmCLA4* (the ortholog of *LAZY1* in rice and Arabidopsis) responsible for multiple phytohormone mediated pathways negatively regulated LA by altering mRNA accumulation. Further analysis showed that ZmCLA4 could directly bind to two key components of BR signaling *ZmBZR3* and *14-3-3*, and two important responsive transcription factors of ABA *ZmWRKY4* and *ZmWRKY72* respectively, thus mediating the crosstalk between BR and ABA in LA regulation [84]. In maize, a bHLH transcription factor *ZmIBH1-1* is a negative regulator of LA in maize. Transcriptome analysis suggested that the *ZmIBH1-1*-mediated LA in the leaf ligular region was highly correlated with cytokinin (CK), JA, and ethylene synthesis and signal transduction pathways associated genes, in particular the two CK responsive genes, GRMZM2G145280 (*BBC1*) and GRMZM2G149952 (*ZmAS1*) that were tightly correlated with cell division, implying that CK may modulate LA through the control of cell profile [85]. Notably, it has been demonstrated that CK indirectly interacted with BR through auxin pathway. For example, BR regulated the development of root primordia through increasing the *PIN* genes expression while the CK inhibited the root primordia by repressing *PIN* genes [86]. However, it was also found that CK was accumulated in the young seedling of wheat with BR treatment [87], whereas overexpression of the rice *Isopentyl Transferase* (*IPT*) driven by the promoter of stress- and maturation-inducible gene, *Senescence-associated Receptor Kinase* (*SARK*), resulted in up-regulation of BR genes, including *DWF4*, *BRI1*, and *BZR1* etc., ultimately leading to higher rice yield during water-stress [88]. These studies further suggest that CK interplays with BR in regulating plant growth and stress response. However, it is still unclear whether and how they cooperated in the LA regulation. As mentioned above, the *bHLH* family generally integrated BR signaling to regulate the LA, such as the *OsILI1* and *OsBLR1*, and thus we proposed that the CK may interplay with BR to regulate LA through the *bHLH*-*BBC1/AS1* module mediated BR signaling. 

In summary, almost all of the regulators modulated leaf angle through the phytohormones-dependent manner (Table 1), however, whether there are unknown components independent on phytohormones, the pathway still remains elusive.

## 7. Future Perspectives

Leaf angle directly influences the shape of plant architecture, consequentially affecting yield. To feed the ever-increasing global population, demand of higher crop yield has triggered the breeders to breed new cultivars that maintain sustainable productivity and yield by dense planting with limited arable lands. Therefore, how to genetically manipulating leaf angle has become one of the most important tasks to be tackled in crop genetic improvement. Until now, the regulatory mechanism underlying leaf angle has been extensively elucidated. However, a few issues are still beyond understanding, preventing the application of specific gene resource in term of crop improvement. To address these issues, the relationship among these genes still needs to be further uncovered, in particular the interaction network. On another hand, knockout or overexpression of these genes generally caused pleiotropic effects on plant growth and development in addition to the LA, such as plant dwarfism and smaller grain. Therefore, identification of interest single nucleotide polymorphisms (SNPs) and/or haplotypes of LA genes could not only be an efficient strategy to extent our knowledge about the relevant regulatory network, but also provide suitable alleles for marker selection breeding. The studies of *ZmLG1* provide an excellent case for bridging the basic research and application. *ZmLG1* was initially identified as a key factor for formation of ligule and auricle in maize and rice, and then regard to be an important player during rice domestication [47,90,91]. Further large-scale genetic analysis revealed that *ZmLG1* was the major QTL controlling leaf angle in maize [92,93,94,95]. Based on these studies, a recent study demonstrated that genetic manipulation of *ZmLG1* gene can significantly increase photosynthetic efficiency and maize yield at higher planting density [96]. Therefore, genome resequencing of larger accessions in crop indeed facilitates deciphering interest haplotypes of LA regulators. Alternatively, generation of novel alleles by CRISPR-mediated gene editing is also a powerful and efficient approach regarding this issue. Nevertheless, it would be fascinating to ask if there are novel regulators involved in LA regulation independent on the phytohormones pathways, which might only modulate LA without pleiotropic effects on other traits. On the other hand, it is also attractive to see whether and how LA coordinates or integrates with other agronomic traits in promoting yield, since yield consists of numerous components, such as plant height and tiller number. Furthermore, manipulating the spatial and temporal expression pattern of LA genes may be also important for timely shaping the plant architecture to achieve optimal photosynthesis, as well as eliminating shade avoidance and neighbor interference.

In conclusion, BR signaling pathway performs along with other plant hormones to form a complex signaling crosstalk to coordinate LA architecture under regular growth condition and in response to environmental stimuli. The well-established regulatory network for LA would provide vast promising targets to be manipulated, no matter through traditional molecular marker-assisted breeding or the gene editing technology, for crop architecture improvement to pursue high production.

## Figures and Tables

**Table 1:** Cloned genes associated with leaf angle in rice and maize.

Pathway	Arabidopsis	Maize/Rice	Functions in Leaf Angle ^1^	Refs
**BR**	*AtCYP90D1* (AT3G13730)	*OsCYP90D2* (LOC_Os01g10040)	Positively regulating leaf angle and related to the BR biosynthesis in rice.	[11,24,25,26]
	*AtDWARF4* (AT3G50660)	*OsDWARF4* (LOC_Os03g12660)	Positively regulating leaf angle and catalyzing C-22 hydroxylation in BR biosynthesis pathway.	[11,24]
	*AtCYP724A1* (AT5G14400)	*OsDWARF11* (LOC_Os04g39430)	Positively regulating leaf angle and catalyzing C-22 hydroxylation in BR biosynthesis pathway.	[23,25]
	*AtBR6OX2* (AT3G30180)	*OsBRD1* (LOC_Os03g40540)	Positively regulating leaf angle and catalyzing BR biosynthesis.	[27,28]
	*AtBR6OX2* (AT3G30180)	*ZmBRD1* (GRMZM2G103773)	Positively regulating leaf angle and catalyzing C-6 oxidation in BR biosynthesis.	[49]
	*AtDWARF1* (AT3G19820)	*OsBRD2* (LOC_Os10g25780)	Positively regulating leaf angle and participating in the complementary pathway of BR synthesis.	[64,66]
	*AtSERK2* (AT1G34210)	*OsBAK1* (LOC_Os08g07760)	Positively regulating leaf angle and mediating BR signal transduction.	[34]
	*AtBRI1* (AT4G39400)	*OsBRI1/OsDWARF61* (LOC_Os01g52050)	Positively regulating leaf angle and stimulating BR signal cascade to regulate organ development by controlling cell division and elongation, but is not necessary for organ initiation.	[71]
	*AtBRI1* (AT4G39400)	*ZmBRI1a* (GRMZM2G048294)	Positively regulating leaf angle.	[46]
	*AtBRI1* (AT4G39400)	*ZmBRI1b* (GRMZM2G449830)		
	*AtBZR2* (AT1G19350)	*OsBZR1* (LOC_Os07g39220)	Positively regulating leaf angle and acting downstream of BR signaling.	[35]
	*AT1G75340* (AT1G75340)	*OsLIC1* (LOC_Os06g49080)	A direct target of OsBZR1 and negatively modulating leaf inclination.	[36]
	*AT2G41710* (AT2G41710)	*OsRLA1/OsSMOS1* (LOC_Os05g32270)	Positively regulating leaf angle and direct downstream of GSK2.	[37,38]
	*AtIBH1* (AT2G43060)	*OsIBH1* (LOC_Os04g56500)	Interacting with OsILI1 and negatively regulating leaf angle.	[40]
	*AtIBH1* (AT2G43060)	*ZmIBH1-1* (GRMZM2G388823)	Negative regulator of LA by modulating cell wall lignification and cell elongation in the ligular region.	[85]
	NA NA	*ZmDIL1* (NA)	Positively regulating leaf angle.	[45]
	*AtPRE5* (AT3G28857)	*OsILI1* (LOC_Os04g54900)	Positive control of leaf angle.	[40]
	*AtBS1* (AT1G74500)	*ZmILI1* (GRMZM2G072820)	Positively regulating leaf angle.	[51]
	*AtSPL8* (AT1G02065)	*ZmLG1* (GRMZM2G036297)	Positively regulating leaf angle and directly activating *ZmBRD1* expression, leading to increased BR and leaf angle.	[49]
	*AtNGA1* (AT2G46870)	*ZmRAVL1* (GRMZM2G102059)	Positively regulating leaf angle by regulating *ZmBRD1.*	[49]
	*AtPGP1* (AT2G36910)	*ZmPGP1/ZmBR2* (GRMZM2G315375)	Positively regulating leaf angle and being involved in the polar transport of auxin.	[62]
	*AtGPA1* (AT2G26300)	*OsD1/OsRGA1* (LOC_Os05g26890)	Positively regulating leaf angle by interacting with OsTUD1 to induce *OsBU1*, leading to increasing leaf inclination.	[73,74]
	*AtPUB30* (AT3G49810)	*OsTUD1* (LOC_Os03g13010)	Positively regulating leaf angle.	[73,74]
	*AtKDR* (AT1G26945)	*OsBU1* (LOC_Os06g12210)	Positively regulating leaf angle.	[73,74]
	*AtBIN2* (AT4G18710)	*OsGSK1* (LOC_Os01g10840)	Negatively regulating leaf angle and BR.	[36]
	*AtGSK1* (AT1G06390)	*OsGSK2* (LOC_Os05g11730)	Negatively regulating leaf angle and the expression of downstream BR response genes.	[83]
	*AT1G63100* (AT1G63100)	*OsSMOS2/OsGS6* (LOC_Os06g03710)	Positively regulating leaf angle and BR-mediated signaling pathway.	[83]
	*AtDCL3* (AT3G43920)	*OsDCL3a* (LOC_Os01g68120)	Negatively regulating leaf angle.	[67]
**IAA**	*AtGH3.6* (AT5G54510)	*OsGH3.1/OsLC1* (LOC_Os01g57610)	Positively regulating leaf angle and maintaining auxin homeostasis by catalyzing excess IAA binding to various amino acids.	[58]
	*AtGH3.2* (AT4G37390)	*OsGH3.2* (LOC_Os01g55940)	Positively regulating leaf angle and auxin signal transduction and auxin homeostasis.	[61]
	*AT-MIR393A* (AT2G39885)	*OsmiR393a* (GQ419313.2)	Positively regulating leaf angle but negatively regulating *OsTIR1* and *OsAFB2.*	[59]
	*AT-MIR393B* (AT3G55734)	*OsmiR393b* (LOC_Os04g58400)	Positively regulating leaf angle but negatively regulating *OsTIR1* and *OsAFB2.*	[59]
	*AtTIR1* (AT3G62980)	*OsTIR1* (LOC_Os05g05800)	Negatively regulating leaf angle and being the target of *OsmiR393.*	[59]
	*AtAFB2* (AT3G26810)	*OsAFB2* (LOC_Os04g32460)	Negatively regulating leaf angle and being the OsmiR393 target.	[59]
	*AtIAA17* (AT1G04250)	*OsIAA1* (LOC_Os01g08320)	Positively regulating leaf angle and inhibiting *OsARF11* and *OsARF19*	[60]
	*AtIAA3* (AT1G04240)	*OsIAA12* (LOC_Os03g43410)	Positively regulating leaf angle and auxin signal transduction and homeostasis.	[61]
	*AT5G11430* (AT5G11430)	*OsLC3* (LOC_Os06g39480)	Negatively regulating leaf angle and inhibiting expressions of *OsIAA12* and *OsGH3.2.*	[61]
	*AT5G63830* (AT5G63830)	*OsLIP1* (LOC_Os10g37640)	Negatively regulating leaf angle and interacting with LC3 to inhibit *OsIAA12* and *OsGH3*.*2.*	[61]
	*AtARF2* (AT5G62000)	*OsARF1* (LOC_Os11g32110)	Negatively regulating leaf angle and inhibited by OsIAA1 in the absence of auxin.	[69,70]
	*AtARF5* (AT1G19850)	*OsARF11* (LOC_Os04g56850)	Positively regulating leaf angle and suppressed by OsIAA1.	[60]
	*AtARF19* (AT1G19220)	*OsARF19* (LOC_Os06g48950)	Positively regulating leaf angle and *OsGH3-5* and *OsBRI1,* and affecting the elongation of rice basal internodes and leaves by regulating cell elongation.	[60]
**GA**	*AtSPY* (AT3G11540)	*OsSPY* (LOC_Os08g44510)	Negatively regulating leaf angle and GA. Involving in BR signal transduction and control of the suppression of *SLR1*.	[64]
	*AtGAI* (AT1G14920)	*OsSLR1* (LOC_Os03g49990)	Negatively regulating leaf angle and GA signal transduction.	[64,66]
	*AtGASA4* (AT5G15230)	*OsGSR1* (LOC_Os06g15620)	Positively regulating leaf angle and induced by *OsSLR1*. Directly binding to OsBRD2 to enhance the BR biosynthesis.	[64,66]
	*miR396* (AT2G10606)	*OsmiR396d* (LOC_Os04g57830)	Positively regulating leaf angle and BR-mediated signaling pathway.	[68]
	*AtARF5* (AT3G13960)	*OsGRF4* (LOC_Os02g47280)	Negatively regulating leaf angle and cell enlargement and number, and being the *OsmiR396* target	[68]
	*AtGFP1* (AT2G22840)	*OsGRF6* (LOC_Os03g51970)	Negatively regulating leaf angle and being the *OsmiR396* target.	[68]
	*AtGID1C* (AT5G27320)	*OsGID1* (LOC_Os05g33730)	Positively regulating leaf angle and mediating GA signaling in rice.	[89]
**JA**	*AtJAR1/AtGH3.11* (AT2G46370)	*OsGH3.5/OsJAR1* (LOC_Os05g50890)	Positively regulating leaf angle and being regulated by OsARF19. Redundant with other OsGH3.	[71]
**Ethylene**	*AtACS6* (AT4G11280)	*ZmACS7* (GRMZM5G894619)	Positively regulating leaf angle.	[76]
**CK**	*AtBBC1* (AT3G49010)	*ZmBBC1* (GRMZM2G145280)	Positively regulating leaf angle and cell division.	[85]
	*AtAPS3* (AT4G14680)	*ZmAS1* (GRMZM2G149952)	Positively regulating leaf angle and cell division.	[85]
**SLs**	*AtMAX2* (AT2G42620)	*OsD3* (LOC_Os06g06050)	Negatively regulating leaf angle and related to the SL signaling in rice.	[9]
	*AtD14* (AT3G03990)	*OsD14* (LOC_Os03g10620)	Negatively regulating leaf angle and dual function as a receptor and deactivator of bioactive SLs, related to the SL signaling in rice.	[78]
	*AtCCD8* (AT4G32810)	*OsD10* (LOC_Os01g54270)	Negatively regulating leaf angle and encode carotenoid cleavage dioxygenase (CCD) 8 related to the SL biosynthesis in rice.	[78]
	*AtCCD7* (AT2G44990)	*OsD17* (LOC_Os04g46470)	Negatively regulating leaf angle and encode carotenoid cleavage dioxygenase (CCD) 7 related to the SL biosynthesis in rice.	[78]
	*AtD27* (AT1G03055)	*OsD27* (Os11g0587000)	Negatively regulating leaf angle and encode β-carotene isomerase, related to the SL biosynthesis in rice.	[78]

^1^ Role of the corresponding gene in regulating leaf angle in rice or maize. NA, none available.

## Abbreviations

LA	Leaf angle
BR	Brassinosteroid
IAA	Indoleacetic acid
GA	Gibberellin
SLs	Strigolactones
JA	Jasmonic acid
ABA	Abscisic acid
